# Prenatal ultrasound diagnosis of fetal giant pericardial cyst: A case report

**DOI:** 10.1097/MD.0000000000034119

**Published:** 2023-06-23

**Authors:** Xiaoqing Wang, Weixia Zhang, Mijie Wang, Ronghong Jiao

**Affiliations:** a Hebei Medical University, Shijiazhuang, China; b Department of Ultrasound, Hebei General Hospital, Shijiazhuang, China.

**Keywords:** fetal echocardiography, fetus, pericardial cyst, prenatal

## Abstract

We describe 1 case of fetal giant pericardial cyst was diagnosed by fetal echocardiography in the second trimester, and the changes of the cyst were recorded by follow-up observation in the late trimester and after birth. We then review and discuss the knowledge about its diagnosis and treatment.

**Patient concerns:**

A 34-year-old pregnant woman was referred to our hospital because of a diagnosis of a fetal pericardial effusion at 22 5/7 weeks at another hospital.

**Diagnosis:**

Fetal echocardiography revealed an irregular anechoic area in the right side of the fetal right atrium and right ventricle that was closely related to but not communicated with the pericardiumis and suggested fetal pericardial cyst. Fetal cardiothoracic magnetic resonance imaging showed cystic FIESTA signal in the right lung region, with clear boundary, and a seemingly line-like low signal shadow within.

**Interventions:**

Since fetal pericardial cysts keep decreasing in size during maternal pregnancy, follow-up observation measures are taken.

**Outcomes:**

Fetal pericardial cysts disappear on their own 4 months after delivery.

**Lessons:**

Asymptomatic pericardial cysts in the fetal period can be followed up and observed, and intervention is performed only when the cyst rapidly enlarges or ruptures and becomes infected in the fetal or neonatal period. Echocardiography can be used as a first-line detection method for their initial detection and follow-up.

## 1. Introduction

Pericardium cyst is a rare congenital primary mediastinal cyst. We present a case of a giant fetal pericardium cyst, confirmed by magnetic resonance imaging (MRI) and followed up by echocardiography, and discuss its management during the fetal and neonatal period.

## 2. Case report

A 34-year-old pregnant woman, gravida 4, para 2 was referred to our hospital because of a diagnosis of pericardial effusion at 22 5/7 weeks at another hospital and was 28 weeks old at presentation. The external ultrasound report showed that a 6.5 mm fluid dark area beside the right cardiac wall. Her medical and family history was unremarkable. Her first child was normal and developing well.

**Figure 1.**
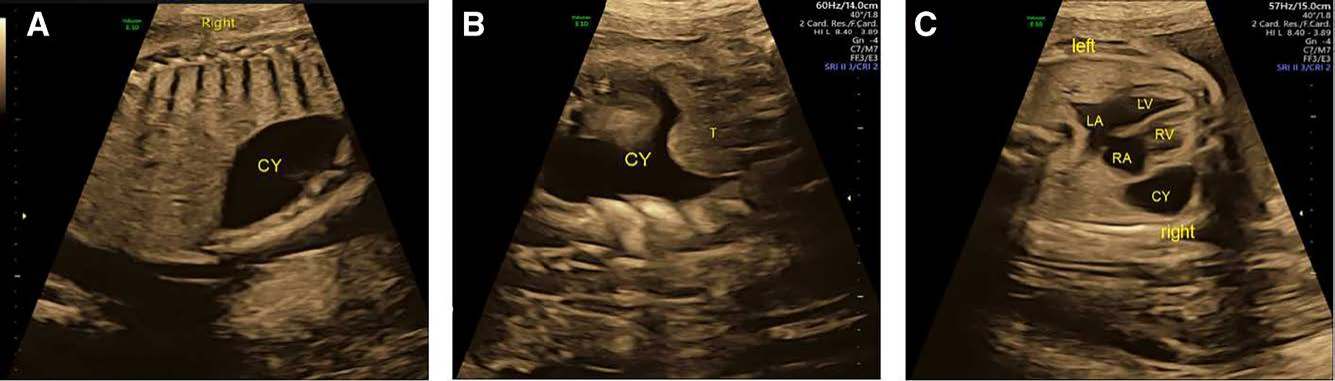
Pericardial cyst. (A) The long diameter of the cyst was shown in sagittal section. (B) The thymus was positioned anterosuperior to the cyst. (C) The 4-chamber view showed that the cyst was on the right side of the right atrium and right ventricle and was not communicating with the ventricle. CY = cyst, LA = left atrium, LV = left ventricle, RA = right atrium, RV = right ventricle, T = thymus.

Applying the instrument GE ultrasound diagnostic machine with a probe frequency of 2 to 5 MHz to make a routine sweeping section of the fetus. Prenatal ultrasound showed fetal growth parameters within normal limits, normal amniotic fluid volume and placenta. Fetal echocardiography showed normal structure and function of the 4-chamber heart, aortic arch, left and right outflow tract, superior and inferior vena cava. The fetal heart rate was neat and moderately strong, about 149 beats per minute. In sagittal section, an anechoic area structure that measured approximately 48 mm × 14 mm × 31 mm in size was detected to the right side of chest, with smooth walls and good internal sound permeability (Fig. 1A). On dynamic scan, the anechoic zone was irregular in shape, located on the right side of the right atrium and right ventricle and was closely related to but not communicated with the pericardium (Fig. 1C). Its anterior upper part was adjacent to the thymus, its upper edge reaches the base of the heart and its lower edge reaches the diaphragm (Fig. 1B). The anechoic area shape had not changed when fetal position changes, it was not affected by the beating of the heart. Moreover, the heart beats well and was not affected by this liquid echo. Color Doppler evaluation of the anechoic zone showed no obvious blood flow signal. Fetal echocardiography suggested fetal pericardial cyst, and she was recommended to review in the third trimester.

**Figure 2.**
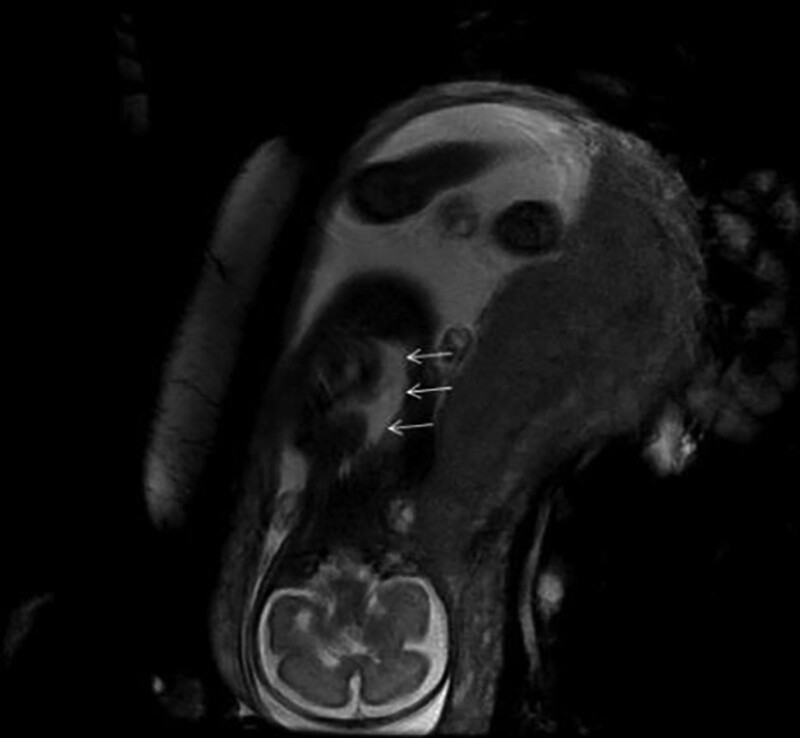
Cystic FIESTA signal was present in the right lung region.

At the same time, the patient completed a fetal cardiothoracic MRI, which showed cystic FIESTA signal in the right lung region, with clear boundary, about 24 mm in length, and a seemingly line-like low signal shadow within (Fig. 2). MRI also suggested the possibility of a pericardial cyst.

**Figure 3.**
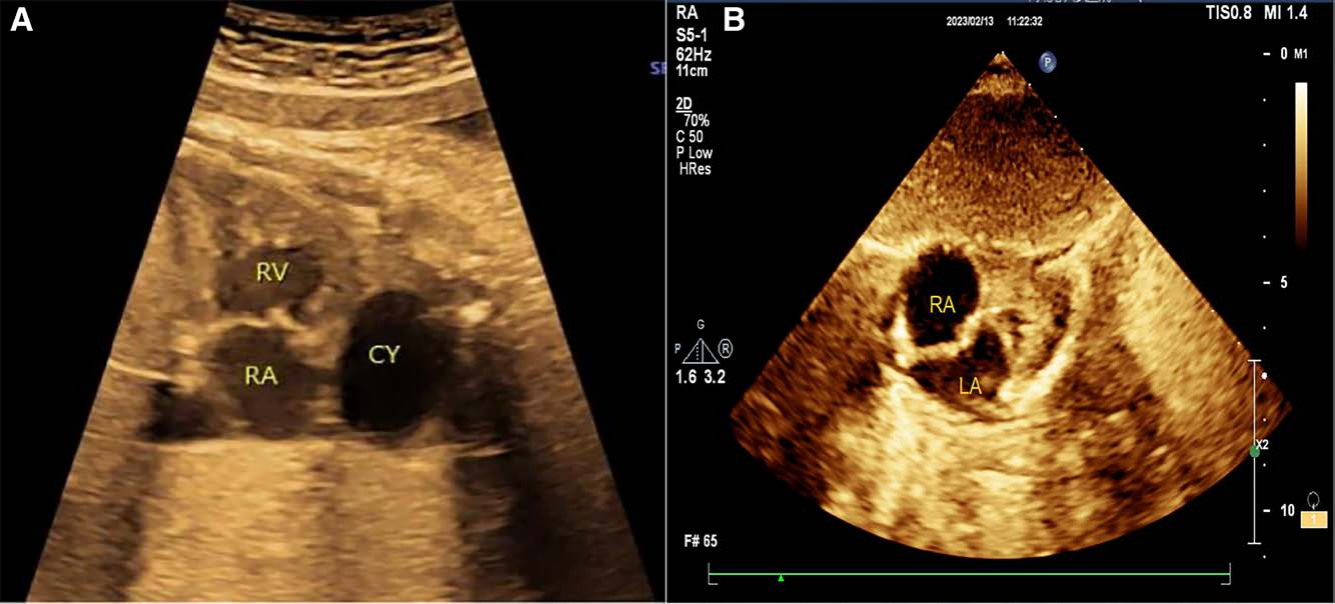
(A) Round cyst located in the right atrioventricular sulcus. (B) The cyst in the right atrioventricular sulcus disappeared at the 4-mo postnatal follow-up.

Thirty-seven at week 1/7, the woman again underwent fetal echocardiography. We found a small circular cyst located in the right atrioventricular groove, anteriorly and upward of the superior vena cava, with a size of 33 mm × 24 mm × 30 mm (Fig. 3A). In this case, considering that the cyst size decreased significantly between 26 and 37 weeks, we presumed that the fetus could be born normally.

At 38 weeks and 2 days of gestation, the mother was delivered by cesarean section due to painful irregular contractions. The newborn weighed approximately 3850 g and had Apgar scores of 10 and 10 at 1 and 5 minutes, respectively.

When the baby came in for a follow-up echocardiogram at 4 months of age, the cyst disappeared and the baby did not have any cardiac or pulmonary symptoms during this time (Fig. 3B). We conclude that the cyst has spontaneously resolved and the prognosis of the infant is good.

## 3. Discussion

Pericardial cysts are rare congenital mediastinal cysts that occur in 1 per 100,000 and account for 7% of all mediastinal tumors.^[1,2]^ Pericardial cysts are caused by incomplete fusion of pericardial mesenchymal Spaces in the embryo and consist of a thin layer of fibrous tissue lined with a single layer of mesothelial cells.^[3]^ On echocardiography, pericardial cysts appear as single or multilocular cystic anechoic structures with thin walls and no obvious blood flow signal. They are most common in the right costophrenic Angle, followed by the left costophrenic Angle, and rarely located in other parts of the mediastinum.^[4,5]^ Adult patients are usually asymptomatic and are found incidentally during chest imaging.^[6]^ Symptomatic patients may present with chest pain, palpitation or dyspnea depending on the location and size of the cyst. Serious complications such as cardiac tamponade, right ventricular outflow tract obstruction or sudden death may also occur when the cyst ruptures, bleeds or compresses other structures.^[7]^

Pericardial cysts in pregnancy are very rare in routine investigations. This is the largest reported case of a fetal pericardial cyst diagnosed by prenatal ultrasound so far. In addition to pericardial cysts, cystic lesions near the fetal heart can also be bronchial cysts, lymphangioma, neural tube gastrulation cysts, and localized pericardial effusion.^[8]^ These lesions should therefore be part of the differential diagnosis of fetal pericardial cysts. Mediastinal bronchial cysts are usually small, located near the carina between the trachea and esophagus, and can cause respiratory distress in the fetus after birth.^[9]^ Neural tube gastrulation cysts are mostly located in the right posterior thorax and often accompanied by fetal vertebral abnormalities.^[10]^ Congenital thymic cysts are rare, which are caused by the abnormal decline of the gill sac tissue in the pharynx during embryonic period and often located in the anterior mediastinum.^[11]^ The depth of pericardial effusion can vary with cardiac systole and diastole. Fetal paracardiac lymphangioma is a cystic malformation of lymphatic vessels, often multiple or multilocular.^[10]^ There are reports in the literature of fetal pericardial cysts with suture-like pericardial effusion visible on both sides.^[8]^

The reported cases of fetal pericardial cyst diagnosed by prenatal ultrasound to date are usually not combined with other anomalies and abnormal signs, and only a few cases of fetal tachycardia have been reported, but the symptoms disappear after birth.^[8,10,12,13]^ Follow-up is recommended for asymptomatic lesions in the neonatal period. Surgical intervention is recommended only when symptoms appear in the fetal or neonatal period or the cyst increases rapidly and ruptures.^[10,14,15]^

## 4. Conclusion

In this case, the diagnosis of pericardial cyst was based on imaging instead of histology. Pericardial cysts are irregular in shape, and the measurement of maximum diameter does not correctly reflect the overall measurement of pericardial cysts. Imaging by magnetic resonance and computerized tomography were particularly useful to diagnosis the pericardial cyst. Echocardiography can show the location, size, morphological changes and internal echo of pericardial cysts, as well as the relationship between pericardial cysts and surrounding tissues, as well as the compression of surrounding tissues, which can be used as the first-line examination method for initial discovery and follow-up observation.

## Author contributions

**Writing – original draft:** Xiaoqing Wang, Ronghong Jiao.

**Writing – review & editing:** Weixia Zhang, Mijie Wang, Ronghong Jiao.
